# Investigating the Synthesis and Characteristics of UV-Cured Bio-Based Epoxy Vegetable Oil-Lignin Composites Mediated by Structure-Directing Agents

**DOI:** 10.3390/polym15020439

**Published:** 2023-01-13

**Authors:** Brindusa Balanuca, Raluca Sanda Komartin, Madalina Ioana Necolau, Celina Maria Damian, Raluca Stan

**Affiliations:** 1 Department of Organic Chemistry “C. Nenitescu”, University Politehnica of Bucharest, 1-7 Polizu Street, 011061 Bucharest, Romania; 2 Advanced Polymer Materials Group, University Politehnica of Bucharest, 1-7 Polizu Street, 011061 Bucharest, Romania

**Keywords:** *Lallemantia iberica* oil, epoxidized vegetable oil, epoxy-functionalized lignin, bio-based composites, thermo-mechanical properties

## Abstract

Bio-based composites were developed from the epoxy derivatives of *Lallemantia iberica* oil and kraft lignin (ELALO and EpLnK), using UV radiation as a low energy consumption tool for the oxiranes reaction. To avoid the filler sedimentation or its inhomogeneous distribution in the oil matrix, different structure-directing agents (SDA) were employed: 1,3:2,4-dibenzylidene-D-sorbitol (DBS), 12-hydroxystearic acid (HSA) and sorbitan monostearate (Span 60). The SDA and EpLnK effect upon the ELALO-based formulations, their curing reaction and the performance of the resulting materials were investigated. Fourier-transform Infrared Spectrometry (FTIR) indicates different modes of molecular arrangement through H bonds for the initial ELALO-SDA or ELALO-SDA-EpLnK systems, also confirming the epoxy group’s reaction through the cationic mechanism for the final composites. Gel fraction measurements validate the significant conversion of the epoxides for those materials containing SDAs or 1% EpLnK; an increased EpLnK amount (5%), with or without SDA addition, conduced to an inefficient polymerization process, with the UV radiation being partially absorbed by the filler. Thermo-gravimetric and dynamic-mechanical analyses (TGA and DMA) revealed good properties for the ELALO-based materials. By loading 1% EpLnK, the thermal stability was improved to with 10 °C (for Td3%) and the addition of each SDA differently influenced the Tg values but also gave differences in the glassy and rubbery states when the storage moduli were interrogated, depending on their chemical structures. Water affinity and morphological studies were also carried out.

## 1. Introduction

Polymer composites are well-known materials developed for different applications for many years, due to their great engineering properties that can be tailormade for specific uses and advanced applications with the employment of different additives ((nano)particles, fibres, hybrid molecules) [1,2,3,4]. On the other hand, the development of sustainable bio-based polymeric materials and new strategies designed to counteract issues relating to their mechanical or thermal performance resulted in novel composites and/or hybrids suitable for various industries and represents an important research area. By far, the hottest field is related to epoxy resins from natural sources [5,6,7]. Along these lines, triglycerides from various unsaturated vegetable oils (VO) stood out as raw materials for the development of new sustainable epoxy resins, with remarkable performances. They started to be considered when plastic and waste of petroleum origin became one of the major threats to the ecosystem, as using them is a healthy strategy to ensure renewable-based materials along with a sustainable economy [8,9,10,11]. The greater the unsaturation degree of the triglyceride molecule, the higher the epoxy content of the resulting derivative will be, and thus, those oils with a great number of double bonds on their structure are of interest (e.g., soybean, linseed, rapeseed, sunflower) [11].

The less-exploited oil of *Lallemantia iberica* seeds (LALO), with a high content of α-linolenic acid (C18:3, around 70% from total fatty acids content), represents a very interesting natural source for industrial use [12,13].

*Lignin* is a complex macromolecular compound with a dual character containing hydrophobic aromatic rings and hydrophilic -OH moieties and compatibility with a VO-derived monomer that plays an important role in obtaining performant composites. A recent study reports that composite materials based on Bisphenol A epoxy resin, with 15 and 30% epoxidized lignin derivative harnessed from nonedible industrial waste, exhibit superior mechanical properties as compared to non-functionalized lignin composites [14]. According to other reports, the absorption of surfactants on the lignin surface leads to colloidal lignin microparticles [15].

Recently, we have reported the synthesis of new sustainable composites as corrosion protection layers based on epoxidized linseed oil (ELO) and Kraft lignin (LnK) [16]. The main challenge was improving the homogenous distribution of the filler, achieved by a double curing procedure (UV and thermal).

Considering all this, we employed in our study the epoxidized Lallemantia oil monomer (ELALO) and compatibilized lignin (epoxy functionalized LnK, not. EpLnK), the great amount of the epoxy ring being expected to yield composites with improved properties. Also, the addition of LMWG (low molecular weight organogelators) into polymeric matrices may lead to superior materials by improving mechanical properties, scaffolding, modified rheology, dispersion of fillers, etc. [17,18]. In this regard, to surpass the drawback of EpLnK sedimentation during the composites’ fabrication, three different structure-directing agents (SDA) have been employed in the current research, such as organogelators (1,3:2,4-dibenzylidene-D-sorbitol, DBS and 12-hydroxystearic acid, HSA) and non-ionic surfactants (sorbitan monostearate, Span 60), respectively, in an attempt to increase compatibility between the VO-derived monomer and functionalized lignin, based on the reported results summarized below.

*DBS* is a very efficient low molecular weight gelator for numerous organic solvents and polymer melts. The self-assembled networks formed by DBS are able to enhance the mechanical properties of the polymers due to their ability to modify the kinetics of polymer crystallization and to induce crystal orientation, forming 3D fibrillar networks in the polymer matrix [19,20]. The use of DBS and its derivatives in the photo-polymerization of dental composites increased the monomer conversion rate and produced composites with enhanced strength and reduced shrinkage [21] and the thermal polymerization of styrene-methyl methacrylate (MMA) formulations in the presence of DBS yielded transparent polymers with no macroscopic phase separation, with H-bonding between DBS and the carbonyl moiety in MMA being proven by FTIR experiments [22]. The addition of 0–4% (wt) DBS in conventional epoxy DGEBA (di-glycidyl ether of Bisphenol A) and thermal curing treatments yielded polymers with significantly enhanced hardness and toughness. Although DBS seemed not to interfere or participate in the reaction, the glass transition and thermal degradation temperatures of the samples obtained in the presence of 2–4% (wt) organogelator were improved by 3–10 °C and 2–5 °C, respectively [23]. Therefore, there are good premises for the synthetic strategy selected in the current work, the addition of DBS in the ELALO-EpLnK system being able to induce interactions by H-bonding and π-π stacking with the aromatic lignin rings and -OH groups, and this kind of association also being reported [24,25].

*HSA* is also an efficient oleogelator employed in structuring VOs as replacements for solid fats and as delivery systems for lipid soluble molecules [26]. The photo-cationic polymerization of epoxidized soybean oil (ESO) with 4–9% (wt) HSA leading to biobased composites was reported by Shibata and co-workers [27], and the obtained composites revealed dendritic clusters of HSA nanofibers formed in the crosslinked polymeric matrix, which explain the improvement of mechanical properties with the increase in organogelator content. The association of lignin derivatives with HSA salts has been also exploited in the formulation of lubricating greases [28].

*Nonionic surfactants* (*Span 60, Tween 80*) could be absorbed on the epoxidized lignin particles, and their addition is possible to reduce phase segregation during cationic polymerization of the ELALO monomer. The use of Tween-type surfactants is reported to improve the enzymatic hydrolysis of lignocellulose through association with free lignin moieties and preventing the unproductive adsorption of the enzyme to the lignin [29]. *Span 60* may improve the stability and the drug-loading capacity of lipid-core polymeric nano-capsules based on poly(ε-caprolactone) and a medium chain synthetic triglyceride. This result is explained by dual interactions of the surfactant, both with the lipid core (hydrophobic chain-van der Waals) and hydrogen bonding of the sorbitan moiety with functional groups of the model drug, quercetin [30]. Also, the addition of Span 60 into polystyrene-cellulose nanoparticle composite films in different ratios improves the compatibility and dispersion and has a plasticizing effect on the obtained bio-composite films [31].

To obtain the complex ELALO-EpLnK bio-based composites, a UV-photoinduced epoxy ring-opening was selected using triarylsulfonium salt (THA), one of the most employed compounds to convert the epoxidized vegetable oils into polymeric materials [32]. Cationic UV curing is recommended for its important technological and industrial advantages, especially in the context of global warming-related energy consumption and increased costs, such as not needing atmospheric oxygen inhibition, low polymerization shrinkage, a possible enhancement of the monomer conversion using a “dark-curing reaction” in ambient temperature (after removing the UV light) and additional economic benefits, such as low energy consumption and a short reaction time compared to the thermal procedures, which is conducive to a low environmental impact [32,33,34].

To the best of our knowledge, this is the first laboratory experiment which involves the LALO as a raw material to design polymeric composites, while also being the first attempt to fabricate composites based on ELALO and lignin derivatives. The innovation of this research is highlighted through the proposed synthetic strategy, being an important attempt to surpass the drawback related to the filler sedimentation by just adding small amounts of selected compatible SDAs. Another innovation is the employment of certified oleogelators as SDA for VO monomers to improve the compatibility within the polymeric matrix, between the hydrophobic ELALO and the complex structure of lignin filler.

## 2. Materials and Methods

### 2.1. Materials

*Lallemantia iberica* seed oil (LALO) extracted in a cold-pressing process was acquired from PTG Deutschland, Flurstedt, Germany. Kraft lignin (LnK), epichlorohydrin (Epi) and all other reagents and solvents involved for the chemically modification of the LALO and LnK were purchased from Sigma-Aldrich (subsidiary of Merck KGaA, Darmstadt, Germany) and used as received. Dibenzylidene sorbitol (DBS, Sigma-Aldrich), Sorbitane monostearate (Span 60, Merck, Darmstadt, Germany) and 12-hydroxystearic acid (HSA, Alfa Aesar, subsidiary of Thermo Fisher Scientific, Kandel Germany), employed as structure directing molecules and the photo-initiator triarylsulfonium hexafluoroantimonate salts (mixture with propylen carbonate, THA, Sigma-Aldrich) was used for the composite materials synthesis.

### 2.2. Methods

#### 2.2.1. Epoxidation of the Lallemantia Oil

Prior to being used, LALO was structurally characterized by ^1^H-NMR and FTIR. The obtained fatty acid profile, based on the NMR spectrum [35] was useful to calculate the average molecular weight (Mw ≈ 920 g/mol) and the average number of the double bonds (n ≈ 7 C=C/mol LALO) [36,37].

*^1^H*-*NMR* (*CDCl_3_, TMS, δ in ppm*): 5.33 (–**H**C=C**H**–, all unsaturated fatty acids); 5.24 (C**H**–O–CO, glycerol); 4.25, 4.15 (C**H**_2_–O–CO, glycerol); 2.78 (–CH=CH–C**H**_2_–CH=CH); 2.28 (C**H**_2_–COO); 2.03 (–C**H**_2_–CH=CH_2_); 1.59 (C**H**_2_–CH_2_–COO); 1.29, 1.24 (–(C**H**_2_)n,); 0.95 (terminal C**H**_3_, linolenic acid); 0.86 (terminal C**H**_3_, all fatty acids except linolenic acid).

FTIR (ATR, cm^−1^): 3010 (ν _= C–H_); 2926, 2857 (ν_C–H asim, sim_), 1744 (ν_C=O_); 1654 (ν_C=C_, unsaturated fatty acids); 1455 (δ_CH2_); 1373 (δ_CH3_); 1235, 1166, 1097 (ν_C–O_); 715 (ρ_CH2_).

The epoxidation reaction of the fatty acids double bonds was performed based on previously reported procedure for other vegetable oils, using peracetic acid generated in situ. Based on the ^1^H-NMR spectral information’s, the epoxidation degree of the obtained ELALO derivative was calculated to be ~95% [36,37].

#### 2.2.2. Kraft Lignin Chemical Modification

LnK functionalization was performed adopting a literature reported procedure, with epichlorohydrin (Epi) in alkaline NaOH medium [38]. Briefly, LnK and NaOH solution (LnK:NaOH = 1:3 *wt*/*wt*, 20% NaOH sol.) were stirred for 30 min at room temperature. Epi was gradually added (LnK:Epi = 1:10 *wt*/*wt*) and the mixture was stirred for 30 min (room temperature), then the temperature was set at 50 °C and maintained for 3 h. The post-reaction mass was neutralized (pH 6–7), centrifuged and the solid phase was collected, washed two times with distillated water, dried and used as lignin derivative (EpLnK) together with the ELALO compound to fabricate composite materials.

#### 2.2.3. Synthesis of the ELALO-EpLnK Composites

**Figure 1:**
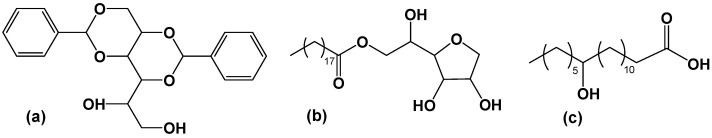
Chemical structure of the used SDA: (**a**) DBS, (**b**) Span 60, (**c**) HAS.

In Table 1, the formulated ELALO-based systems studied within this research are presented. There were tested three different organic SDA agents (1,3:2,4-dibenzylidene-D-sorbitol, DBS; sorbitan monostearate, Span 60; 12-hydroxystearic acid, HSA; chemical structures of the three SDAs are presented in Figure 1), to establish the appropriate molecule with positive influence upon the ELALO-EpLnK blends during the formulation and curing process, in terms of dispersion. Each SDA was added at a concentration of 1% wt. related to ELALO. Two EpLnk concentrations were also tested, 1% and 5%, respectively, calculated based on the amount of ELALO. UV-induced epoxy rings reaction in the presence of THA photo-initiator was chosen for the ELALO-based polymeric composite materials synthesis.

A certain amount of ELALO and SDA was stirred for 15 min at 90 °C, until the visual solubilization of the additive in the oily monomer. Then, the established amount of EpLnK was added, magnetically stirred for 10 min at 90 °C and then, the ELALO-SDA-EpLnK mixtures were sonicated at 50 °C for 20 min, to achieve a good dispersion of the second additive in the ELALO continuous phase. After this step, the beaker was placed into ice bath and maintained for 1 h. At the end, 4% (wt. related to ELALO) THA photo-initiator was added and manually mixed for 5 min. The same procedure was applied for all the studied systems, irrespective of the used additives. A reference sample, without SDA or EpLnK content was prepared (F1).

The resulted mixtures, according to Table 1, were cast in thin films (1 mm) in silicon moulds and cured under UV radiation for 30 min (λ = 365 nm, power = 8 W; the distance between radiation source and sample surface ≈ 5 cm). After UV treatment, the samples were held in the silicon moulds for 24 h and then characterized through different techniques to establish their features and the structure-properties correlation. The main synthesis steps to obtain ELALO-SDA-EpLnK composites are presented in Figure S1 (Supplementary Materials).

### 2.3. Characterization

Nuclear Magnetic Resonance Spectroscopy (NMR). ^1^H-NMR spectra of LALO and ELALO were recorded on a Bruker Advance III Ultrashield Plus 500 MHz spectrometer (Billerica, MA, USA), operating at 11.74 T, corresponding to the resonance frequency of 500.13 MHz for the ^1^H nucleus. Chemical shifts are reported in ppm, using TMS as internal standard.

Fourier Transform Infrared Spectrometry (FTIR). FTIR spectra were registered on a Vertex 70 Brucker (Bruker Scientific LLC, Massachusetts, USA) spectrometer using the Attenuated Total Reflectance (ATR) sampling technique. Sample analysis was run with 32 scans in the wavenumber region 600–4000 cm^−1^, at room temperature.

Gel Fraction determination (GF). The soluble fraction of the studied ELALO-based materials was measured by extraction in tetrahydrofuran (THF). The GF measurements were performed in triplicate; pre-weight samples (weight *w*1) were subjected to THF extraction for 24 h when they were dried to constant weight at 60 °C (weight *w*2). GF was calculated with the Equation (1):

(1) $$\mathrm{GF}\;\left(\%\right)=\frac{\left(w1-w2\right)}{w1}\;\times \;100$$

Water affinity. Static Contact Angle (CA) measurements were performed on DSA100E (KRUSS GMBH, Hamburg, Germany) equipment using Drop shape analysis method. Ultrapure water droplets were used with a drop volume of approximately 2 µL, CA values being registered within 10 s of the drop contacts with materials surface. Three determinations were made for each specimen, at room temperature. *Water Absorption* (*WA*) capacity of the studied composites was determined using the standard water absorption ASTM D570 method. Pre-weighted samples (*m*_0_) were immersed in distilled water (50 mL) for 7 days, when they were taken out, tapped with filter paper to remove excess water and then the final mass was recorded (*m*_1_). WD measurements were done in triplicate for each studied ELALO-based system, the reported values being the average of three results, calculated with using Equation (2):

(2) $$WA\;\left(\%\right)=\frac{\left(m_{1}-m_{0}\right)}{m_{0}}\;\times \;100$$

Dynamic Mechanical Analysis (DMA). Thermo-mechanical properties were measured on a Tritec 2000 instrument (Triton Technology Ltd, Loughborough, UK), operated in single cantilever bending mode. Rectangular materials were tested at 1 Hz frequency, from −40 to 100 °C (5 °C/min heating rate).

Thermo-Gravimetric Analysis (TGA). All synthesized ELALO-based composite materials were investigated by TGA in nitrogen atmosphere, using TG 209 F1 Libra equipment (Netzsch-Gerätebau GmbH, Selb, Germany). Thermal degradation profile was registered in the temperature range 25–700 °C (heating rate 10 °C/min).

Scanning Electron Microscopy (SEM). The morphology of the composite materials was explored in the SEM images registered on Quanta Inspect F device equipped with a field emission gun with a resolution of 1.2 nm. Before being scanned, selected ELALO-EpLnK based composites were broken in liquid nitrogen and sputtered with a thin layer of gold.

X-ray photoelectron spectroscopy (XPS). XPS analysis was made using Thermo Scientific K-Alpha equipment (Thermo Fisher Scientific, Massachusetts, USA), fully integrated, with an AlKα monochromatic source (1486.6 eV). Survey spectra was recorded on full binding energy domain with a pass energy of 200 eV, considering the C1s position at 284.8 eV as internal standard. High resolution O1s spectra was registered with a pass energy of 20 eV. Deconvolution of high resolution O1s spectra was done using a Gaussian-Lorentzian algorithm with convolved stages.

## 3. Results and Discussion

The research strategy starts from the highly unsaturated LALO (n ≈ 7 C=C/ LALO molecule, with more than 64% C18:3 from the total fatty acids [39]) being converted into epoxy monomer (ELALO, epoxidized degree ≈ 95%, calculated from ^1^H-NMR data), before being used to get new sustainable polymer composites by loading compatibilized lignin (lignin bearing epoxy groups, EpLnK, synthesized in the current study). To surpass the issue of the filler dispersion within the oil phase, three SDA have been used: DBS, Span 60 and HSA.

### 3.1. Synthesis and Characterization of the ELALO Monomer and EpLnK Derivative

**Figure 2:**
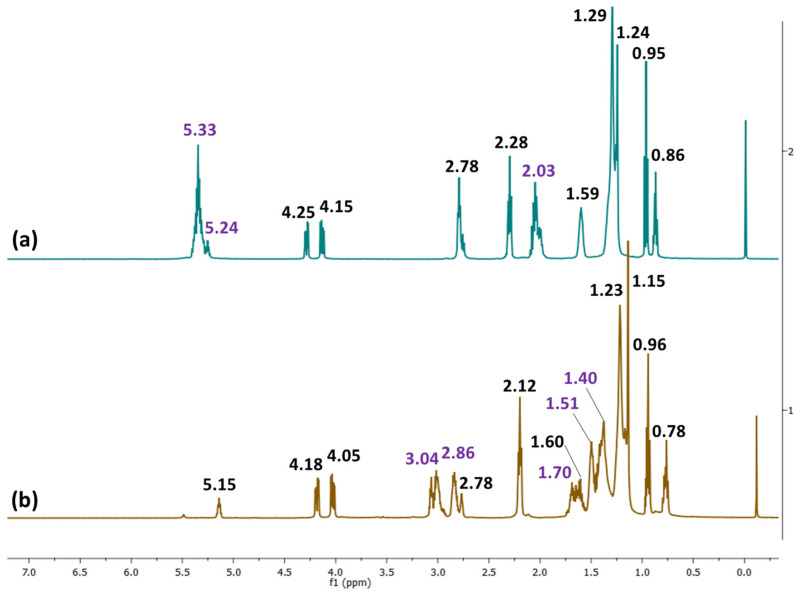
^1^H-NMR spectra of (**a**) crude LALO and (**b**) epoxidized LALO.

***Oil derivative.*** Structural analysis of the ELALO monomer by ^1^H-NMR revealed the success of the epoxidation reaction, with specific signals of those protons from the oxirane rings being noticed, as proven by Figure 2: 3.04 ppm, 2.86 ppm, 1.70 ppm and 1.40 ppm in the Figure 2b spectrum, as compared to spectrum from Figure 2a, where the signal of the double bonds from the fatty acids can be observed at 5.33 ppm and 2.03 ppm [36,40].

**Figure 3:**
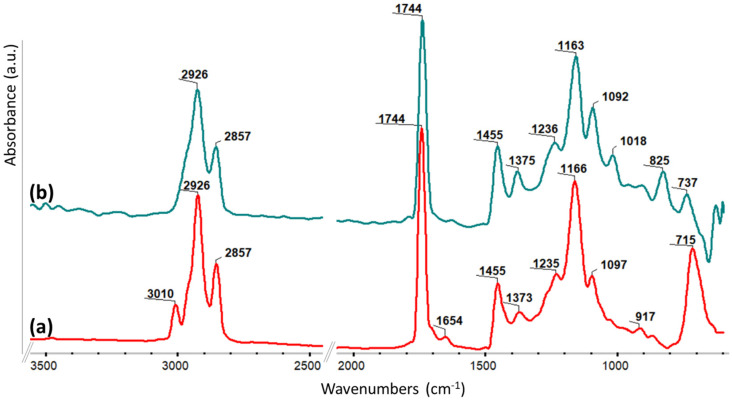
FTIR spectra registered for (**a**) LALO and (**b**) ELALO.

Chemical modification of the LALO structure was also interrogated through FTIR spectrometry, confirming the presence of the epoxy rings on the ELALO derivative through the new absorption bands at the wavelength of 815–960 cm^−1^ (Figure 3b). It was also noticed that the absorption bands at 3010 and 1554 cm^−1^ (ν_Csp2-H_ and ν_C=C_, respectively) in the LALO FTIR spectrum (Figure 3a) have not been registered for ELALO, proving their consumption during the epoxidation reaction [36,41].

***Lignin derivative.*** Lnk was functionalized with epoxy moieties as a tool for improving its compatibility with the continuous oily phase of ELALO and preventing the sedimentation during the UV curing procedure, as reported previously [16].

Structural characterization of the resulting EpLnK was performed using FTIR spectroscopy (Supplementary Materials, Figure S2). Evidence of the oxirane rings was noticed: there is a slight displacement of the absorption bands around 1300–1200 cm^−1^ due to the presence of new C-O bonds (ether), and a remarkable shape of the absorption bands in the spectral region of 1730–1588 cm^−1^ ascribed to the epoxy groups and due to the stimulation of the aromatic nuclei [38,42]. A broad band centred at 3400 cm^−1^ (–OH stretch) and intense bands at 2936 and 2875 cm^−1^ (–CH_2_/–CH_3_ asymmetrical and symmetrical stretching) characterize the lignin spectrum. Conversely, the hydroxyl band in the chemically modified lignin spectrum is shifted to 3200–3300 cm^−1^, in accordance with other reports [15]. Also, the C-H stretching bands (CH_2_ and CH_3_ around 2800–2950 cm^−1^) appear to be shifted and less intense, confirming the lignin structure’s functionalization during the reaction with epichlorohydrin.

The XPS analysis (Supplementary Materials, Figure S3 and Table S1) also indicates the presence of the new oxygenated functional groups of the lignin derivative structure, the overall C-O ratio for the EpLnK being calculated at 1.04% versus 3.44% for the neat LnK. The higher oxygen content in EpLnK was expected, once the epoxides were grafted and part of the -OH groups (LnK) were converted, according to Figure S4 (Supplementary Materials).

### 3.2. Synthesis of the Bio-Based Composites, Structural Analysis and Evaluation of the Curing Protocol

The procedure used to fabricate the ELALO-based materials within this study was adapted in consideration of the physical properties of the employed components, the use of the SDA generating the heating and cooling steps for the sample’s formulation. After the solubilization of DBS, Span 60 and HSA in the VO monomer, the obtained mixtures were cooled to induce gelation, to “freeze” the system in that configuration given by each additive, or to trap lignin molecules within the ELALO-SDA structure, avoiding sedimentation.

#### 3.2.1. SDAs’ Effect on the ELALO Structural Organization

**Figure 4:**
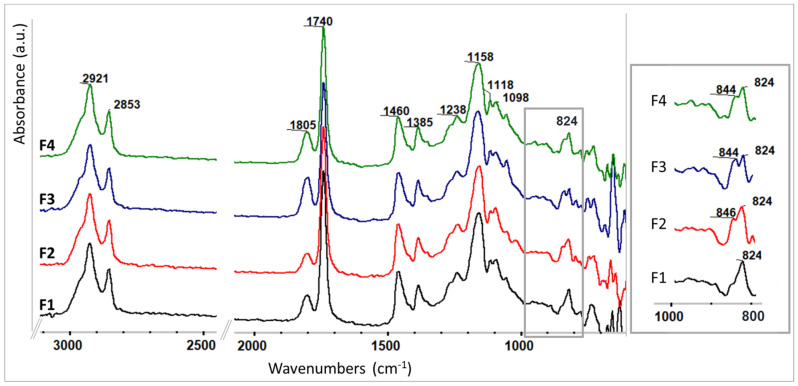
FTIR spectra of the initial (unpolymerized) ELALO and ELALO-SDA systems. Detail—cropped and zoomed spectral region from 1000–800 cm^−1^.

Structural analysis of the formulated ELALO-based initial systems, with or without additives, was performed using FTIR spectroscopy. First, the influence of SDA addition upon the structural arrangements of the studied formulations was investigated and Figure 4 presents the overlapped spectra of the F1–F4 unpolymerized systems. No major difference was observed when F2–F4 spectra were compared to the reference system, F1. Considering the chemical structures of ELALO, lignin and SDA, we presume H-bonding to be the main driving force in association with the monomer and additives before the UV exposure. The 1805 cm^−1^ absorption band and the soft splitting of the 1092 cm^−1^ (ELALO spectrum—Figure 3b) in 1118 and 1098 cm^−1^ picks are assigned to the propylene carbonate from the photo-initiating system. Small differences can be observed in the spectral range 850–820 cm^−1^ for F2-F4 systems, characteristic of the epoxy rings in EVO. That maximum at 824 cm^−1^ depicts a supplementary band at 844/846 cm^−1^ and may be attributed to an associated epoxy ring with hydroxyl groups from SDA molecules via H-bonding. This hypothesis is sustained by the reported literature results, such as an increase in the reactivity of the curing systems due to initial H-bonding between epoxy group and amines or β-hydroxyamines being used as curing agents [43], or the demonstration of an intramolecular H-bonding between the -OH group and epoxy in the *cis-*diasteroisomer of 1,2-dialkyl-2,3-epoxycyclopentanol and, by consequence, a higher stability than the *trans-*diasteromer using the M06-2X 9 density functional theory (DFT) method [44]. A positive shift of the C-O stretching vibration was reported in all the cases involving H-bonding, including, for example, the absorption of several organic molecules on graphene oxide [45]. Additional information was obtained by examining the modification of the absorption bands attributed to the C=O stretching vibration from the ELALO in the presence of the SDA and lignin derivative, presented in Table 2.

As the literature mentions, the presence of H-bonding involving C=O groups may be evidenced using FTIR spectroscopy through the observed shifts towards lower wavenumbers of the carbonyl group stretching band around 1740 cm^−1^ [46]. All the SDA molecules and the filler lignin involved in this study contain -OH groups with a significant contribution in the auto-association that are supposed to contribute to the ELALO structural organization. The data presented in Table 2 sustained this hypothesis, with a shift in the C=O band being observed for all the samples containing additives.

For DBS, H-bonding is the main interaction conducing to fibril structures and this tendency is extended to DBS-MMA systems, when the formation of H bonds with C=O ester bonds was observed [22]. Moreover, in the particular case of ELALO, due to the special structure of the oil with a high unsaturation degree almost fully converted to epoxy rings, it is expected that H-bonds will also form with the additional oxygen atoms, as discussed above.

When Span 60 is used, due to the high compatibility of this additive with the ELALO monomer, the hydrophobic chain of the fatty acid ester of sorbitan settles preferentially, probably along the fatty acid chains in the epoxidized oil. Thus, a great influence is exerted due to the H bonds interactions of the sorbitan rings from Span with the epoxy rings from ELALO, as evidenced in the FTIR spectrum through the splitting of the characteristic epoxy band at 824 cm^−1^. HSA self-association is also achieved through H-bonding, but, in this case, a linear structure is involved. The structural similarity with ELALO causes the H bonds from -OH (HSA) to be formed with the accessible epoxy rings for the F4 formulation. We assume that the increased intensity of the 844 cm^−1^ band (Figure 4—zoomed spectral region) may be explained by a large amount of H-bonding with the epoxy groups.

#### 3.2.2. The Influence of the SDA Addition upon the Polymerization Process

**Figure 5:**
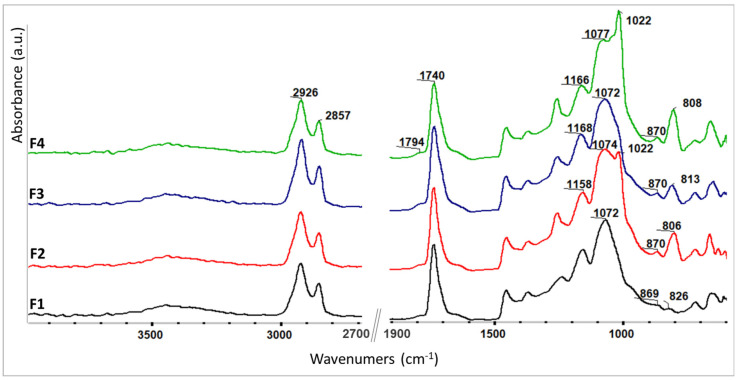
FTIR spectra of the final F1–F4 materials (after UV treatment).

The epoxy ring opening reaction involved in the polymerization process was monitored using FTIR spectroscopy. For the final F1–F4 ELALO-SDA-type materials, Figure 5 presents the registered FTIR spectra. We noticed new absorption bands in the 1022–1077 cm^−1^ spectral region, attributed to different stretching and bending vibrations of the C-C-O, C-O and O-H bonds, formed during the photo-polymerization reaction, according to the cationic UV curing mechanism of the epoxy rings [32,33].

The efficiency of UV-curing may also be estimated by examining the spectral range of the C-O stretching bands presented in Figure 5. According to the literature reports, the C-O stretching vibration in the range of 1156–1168 cm^−1^ is attributed to ether bonds in the triglyceride ester bond of epoxidized vegetable oil (designated as “Ether” A) unchanged during UV-curing, while the band around 1072–1077 cm^−1^ (designated as “Ether B”) is attributed to the C-O bonds formed by ring-opened epoxide groups after the UV-curing process [47]. According to the relative intensity of these bands, taking “ether A” as a reference, one may conclude that the conversion of epoxy rings after UV-curing was comparable for all the F1–F4 materials, a conclusion sustained by the determined GF fraction (Table 3).

The opening of the epoxy rings during UV curing is proven also by the increased intensity of the O-H stretching vibrations (3500 cm^−1^). The absorption bands around 770–900 cm^−1^ are presumably due to aliphatic and aromatic C-H and C=C vibrations from the used components (monomer, SDA, THA).

#### 3.2.3. The Influence of the EpLnK and SDA upon the Polymerization Process

**Figure 6:**
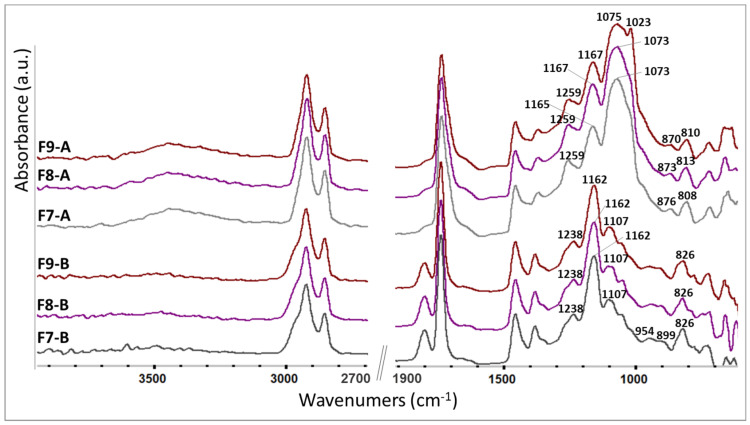
FTIR spectra of the initial and final F7–F9 composites. (Before curing—not. B; after curing—not. A).

For the initial (unpolymerized) F7-F9 ELALO-based systems (1% EpLnK, different SDA, Figure 6), the FTIR spectra show similar profiles with those registered for lignin-free systems (F1–F4), but when lignin-containing final materials (cured) were investigated, structural differences could be observed for the different incorporated SDA. Those samples containing DBS and Span60 (F7-a and F8-a spectra) show an intense envelope centered at 1073 cm^−1^, attributed to the “ether B” resulting after epoxy ring opening (see above) while F9 materials (containing HSA) indicates a second peak located at 1023 cm^−1^, which may be assigned to the EpLnK placed near the surface, an affirmation sustained by a higher hydrophilicity, as determined by the contact angle measurements (Table 3).

Spectral changes in the epoxy and hydroxyl characteristic ranges for all the ELALO-EpLnK composites (Figure 6, F7 A–F9 A) sustained the success of the UV curing treatment.

#### 3.2.4. Conversion of the Epoxy Groups by GF Measurements

The calculated GF values (Table 3) support the strategy of using SDA to obtain ELALO-EpLnK composite materials for all the SDA experiments, as comparable conversions were obtained (F1–F4). The most significant variation in the GF values was observed for the ELALO-EpLnK5% composites (F10–F12), irrespective of the nature of SDA employed, and there was a decrease in the epoxy conversion of 20% for HSA, 17% for DBS and 13% for Span 60. An explanation for his behaviour may be the well-known potential of lignin to be a UV blocker [48], with an increased concentration of EpLnK affecting the success of UV curing. However, the use of Span60 (F3, F8, F11) may be considered as the most promising SDA in the case of vegetable oil-lignin composites, even for higher lignin derivative concentrations.

### 3.3. Water Affinity of the ELALO-Based Materials

Surface and bulk wetting properties were investigated through contact angle (CA) and water absorption (WA, at 24 h) measurements (Table 3). These are important features associated to the large potential applications of the designed materials (coatings, adhesives, substrates/ encapsulation systems for electronics etc), the additives involved in the fabrication strategy being able to influence the affinity for water in one way or another. The CA values are directly influenced by the SDA chemical nature, with Span 60 conducting to the more hydrophobic surface material as compared to the neat ELALO (~103° for F3—ELALO-Span1 and 84° for F1—ELALO, respectively), while DBS led to the more hydrophilic one, regarding the ELALO-SDA-type materials. When EpLnK was loaded (without SDA addition), 10° higher CA and 15° lower CA were obtained for the 1% and 5% additive concentration, respectively. This behaviour is related to the lignin structure bearing both hydrophilic and hydrophobic units, but it could also be related to the density of the reaction/polymerization points within the oil-based matrices, the lower conversion degree (GF) or the higher possibility of water penetration through the mesh of the network.

The CA results are in good agreement with the calculated WAD, with ELALO-Span-type systems being the more hydrophobic materials from the category to which they belong (with one or both additives in different concentration), correlated with the Span structure, bearing the C18 long fatty acid chain.

### 3.4. Thermo-Mechanical Properties

**Figure 7:**
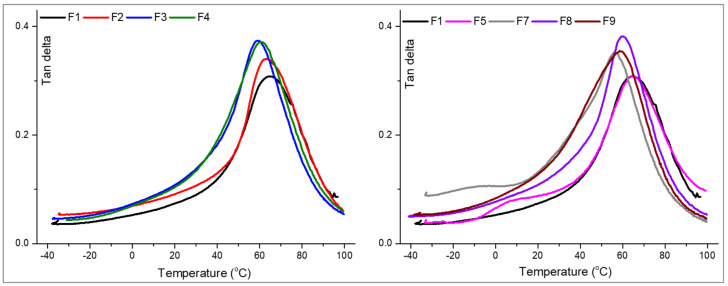
DMA curves (Tan delta vs. temperature) recorder for selected ELALO-based materials. Influence of the SDA—left graph and influence of both additives—right graph.

Dynamic-mechanical analysis was used to investigate the possible influences of the used additives under the ELALO features. From the registered DMA curves (Figure 7), it can be observed that Span and HSA slightly decrease the Tg values in accordance with their chemical structure with long carbon chains. On the other hand, the addition of the EpLnK does not appear to have a significant effect on the ELALO thermo-mechanical features, probably due to the small concentration used. When both additives were used to synthesize the composite materials, the same small influence of these molecules was noticed. In the context of the SDA addition, this is a favourable behaviour, considering the long chains of the Span60 and HSA which do not dramatically decrease the Tg values.

On the other hand, DMA results indicate that EpLnK does not act as a reinforcing agent, but the great potential of the LnK and its derivatives in different applications (such as anticorrosive protection, electronics industry, and many others [16,49,50]) compensates for this inert behaviour in terms of rigidity and thermo-mechanical resistance. The overall behaviour of the studied ELALO-SDA-EpLnK materials, as well as the shape of the registered curves, with a single maximum associated to the Tg by Tanδ peak, may talk about homogeneous materials, the purpose associated with the SDA addition being thus achieved.

For F5 sample (ELALO-EpLnK) a second transition was observed around 10 °C. According to the DMA theory, this β relaxation may be associated to the motion of some segment (4–6 carbon atoms or) from the main chains. The Tanδ peak augmentation may also be associated with a heterogeneous material, in terms of different domains with high or reduce number of reaction points, due to the multifunctional monomer and lignin derivative; such a curve shape could also be associated with a high crosslink degree (transposed as a high polymerization degree). In this context, corroborated by the calculated GF values indicating great conversion of the epoxy rings for F5 (ELALO-EpLnK1, GF ~ 96%), this β transition at 10 °C may not be due to unreacted species, but may be due to the large volume molecule of the lignin derivative creating internal space and consequently influencing the movement for those segments of the ELALO outside the reaction centres (segments C2–C8, C11–C18 or C14–C18, depending on the fatty acid, C18:3, C18:2 or C18:1). A small transition at negative temperature was also observed for F7 material, containing EpLnK and DBS. This may be explained by a possible π-π stacking association of DBS aromatic rings with lignin, thus increasing the free volume within the 3D network.

Looking at the obtained DMA graphs for EpLnK-loaded F8 and F9 materials (ELALO-Span1-EpLnK1 and ELALO-HSA1-EpLnK1), the Tg does not dramatically decrease, the Tanδ peaks are wider and the absence of any secondary transitions was noticed. It is a possible proof supporting their role in the achievement of a homogeneous polymeric assembly mediating and organizing the systems’ micro-structure, conducing to a great number of reaction centres transposed in a great polymerization degree, according to the GF measurements.

A storage modulus is used as a measure of the elastic response for an accurate overview of the studied materials. The obtained data indicate that both additives (SDAs and EpLnK) exert influence on viscoelastic properties of the studied complex systems. The measured frequency was fixed at 1 Hz. The DMA results (Table 4 and Figure S5) show that the storage modulus decrease gradually with increase of the temperature in the glassy region, until a sharp decrease attributed to the glass transition temperature (Tg) above which, the epoxy network is in the rubbery region. The ELALO-EpLnK1 material (F5) appears as the most flexible sample according to the lowest storage modulus values from the glassy state while the neat ELALO polymer network (F1) is the stiffest one. These findings could be an indicator of the homogeneity established in the final networks, thus F1 sample being a constant ELALO network while in the case of F5 composite, microphases of EpLnK may be form, creating a heterogeneous effect of different phases. However, these phases are not considered to be crystalline because of the broad transition from a glassy to a rubbery state, which, according to [51], is attributed to amorphous polymer networks.

When the samples are looked at in the glassy state (Table 4), the improvement of the storage modulus values with the addition of the SDA agent in the presence of EpLnk can be noticed in the case of all three molecules. Thus, when comparing the F2 and F7 samples (ELALO-DBS1 and ELALO-DBS1-EpLnK1, respectively), it can be observed that DBS generate a stiff material, highlighting the good influence of EpLnK (π-π stacking) upon this complex ELALO-SDA system, with an increase of the storage modulus of 2400 MPa.

The same improvement of the storage modulus with 2000 MPa was noted also for F4 vs. F9 composites (ELALO-HSA1 and ELALOHSA1-EpLnK1). The slight deviation of the associated Span 60 behaviour, reflected in a smaller increase for the storage modulus in the ELALO-Span1-EpLnK1 composite (with 1600 MPa), when compared to ELALO-Span1 and) in the ELALO composite, is probably due to the flexible structure giving a certain mobility to the final network, as was observed from the Tanδ results. This behaviour is assigned to an improvement in glassy state stiffness in the complex bio-based systems. Additionally, in the case of using DBS, it is possible that this additive induces the nucleation of crystallization, which could explain the sharp transition from a glassy to a rubbery state, according to Figure S5.

When the samples are reaching the rubbery state, the presence of β-relaxation observed for F5 (ELALO-EpLnK1) and F7 (ELALO-DBS1-EpLnK1) in the Tanδ curves can be explained through the micro-phasic supramolecular structure given by the presence of the EpLnK particles in this particular case. The high storage modulus values in the rubbery state for the ELALO-EpLnK containing Span (F8) and HSA (F9) additives suggests an anti-plasticizer effect of these molecules, as defined in [51]; the flexible chains could fill the free volume from the EpLnK phase and restrict the local-mode molecular motions associated with the β-relaxation.

### 3.5. Thermal Stability of the ELALO-Based Materials

Thermal stability was not dramatically affected by the SDA molecules at the initial phase of the experiment. But Span 60 leads to a slight decrease of the thermal properties in this first heating range. As the experiment progresses, a noticeable but not higher decrease of the thermal stability was observed for the ELALO-SDA materials, Span 60 being the additive that stands out through this negative influence (Td at 10% mass loss decreases with ~5%, comparing to the neat ELALO sample—Table 5).

Loading EpLnK particle, an improved thermal degradation profile was observed for F5 and F6 for the whole experiment temperature range, argued by the grafted epoxy rings participating to the 3D structure and, on the other hand, to the aromatic structure of the filler. We remark that the integration of the compatibilized LnK within the continuous phase of epoxidized oil could be an advantage, associated to a better dispersion when compared to the addition of the neat LnK which conducted to a non-satisfying dispersion within the epoxy linseed oil matrix, as we reported elsewhere [16].

Loading SDA within ELALO-EpLnK composites produces an improvement of the thermal stability, probably due to their orientation effect on the ELALO macromolecules and lignin particles, respectively, leading to homogeneous systems. The F8 sample, containing Span 60, seems to be a better ELALO-derived system containing lignin derivative. At temperature above 350 °C, all the studied ELALO-based materials registered a similar decomposition profile. Even if thermal properties are improved when 5% EpLnK is loaded, with the negative influence upon the epoxy ring opening (reflected to GF values), this effect can only be attributed to the additive ratio and is less accountable for a network with a larger number of reaction points.

### 3.6. Morphological Investigation of the ELALO-Based Composites

**Figure 8:**
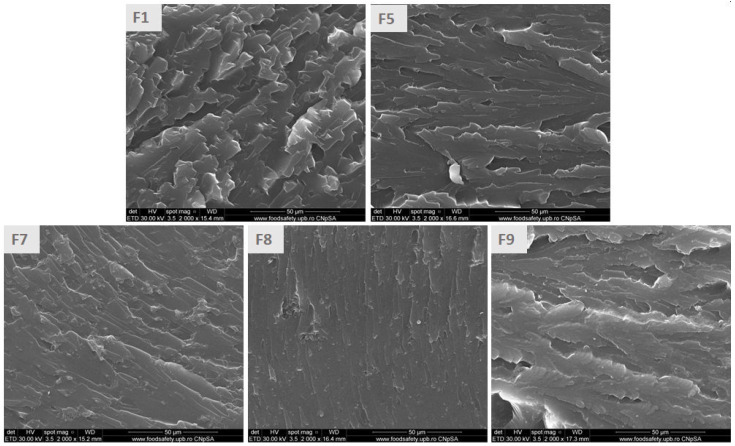
SEM images for different ELALO-based materials (ELALO (**F1**), ELALO-EpLnK1 (**F5**), ELALO-DBS-EpLnK1 (**F7**), ELALO-Span-EpLnK1 (**F8**), ELALO-HSA-EpLnK1 (**F9**); 2000× magnification).

When composite materials were investigated regarding their morphology, the addition of the selected SDA was noticed. According to Figure 8, F1, representing the neat epoxy matrix (ELALO), shows the characteristic architecture of polymers derived from epoxidized vegetable oils [37], with a puzzle-like structure. ELALO-EpLnK based composites with SDA content look very different, depending on the additive involved in the synthesis. Span 60 led to the smoothest material (F8), while the HSA organogelator conducts to a rough architecture (F9), this behaviour being also observed for canola oil and HSA (2%) organogels [52].

### 3.7. XPS of Selected Composites

The XPS survey spectra were recorded for ELALO-Span-EpLnK-type composites, which were selected based on the SEM images showing a smooth surface, a sign of a good homogeneity. The freshly cut surface was subjected to analysis, and the recorded data revealed a small influence of 1% Span addition in the ELALO (for F3 material) on the C/O ratio according to Table 6. This remark may be explained by the small concentration of the additive bearing oxygen atoms which may be involved in some interaction with the oily monomer during the formulation and photo-polymerization steps. In the ELALO-EpLnK1 composite material, an increase of the C/O ratio was observed due to the large number of oxygen atoms from the lignin derivative and then, when Span60 was loaded, the C/O ratio calculated for F8 material (ELALO-Span1-EpLnK1) slightly increased due to the presence of both additives. These C/O values are in accordance with the thermo-mechanical properties which showed the influence of Span 60 on the compatibility between EpLnk and ELALO matrix, through a highlight given on the importance of functional groups within the overall composite network structure.

## 4. Conclusions

We have investigated new epoxy-based composite materials from highly unsaturated *Lallemantia Iberica* oil and lignin (ELALO and EpLnK) obtained by sustainable cationic UV photo-polymerization procedure. The experiments were conducted in order to establish the influence of a structure-directing agent (SDA) upon the epoxy ring conversion and dispersion of the lignin derivative, which is significant to the final materials’ functional properties (morphology, thermal, thermo-mechanical properties, etc). FTIR investigation showed interactions between epoxy monomer and SDA that were in good agreement with the gel fraction measurements.

All the ELALO-based formulations with 1% EpLnK irrespective of the SDA present better thermal stability as compared to neat ELALO or ELALO-SDA materials. The UV blocker potential of lignin significantly decrease the conversion of the epoxides for 5% EpLnK. Using SDA as additive for ELALO_EpLnK composites significantly prevents EpLnK sedimentation, more homogeneous morphology being determined by SEM. As mentioned before, previously attempts for VO epoxy monomer-lignin composites were conducting a dual curing procedure to inhibit the lignin sedimentation.

The most promising SDA proved to be Span 60, the synthesized composites exhibiting an increased flexibility as determined by DMA attempts, increased hydrophobicity, and low water absorption capacity. These are important features for further applications such as coatings, adhesives, electronics industry applications, etc.

## Figures and Tables

**Table 1:** Feed composition of the ELALO-based formulated systems.

Samples Code	Sample Description	Additives			
		EpLnK ^a^	SDA ^b^		
			DBS ^c^	Span 60 ^d^	HSA ^e^
F1	ELALO	0	0	0	0
F2	ELALO-DBS	0	1%	0	0
F3	ELALO-Span	0	0	1%	0
F4	ELALO-HSA	0	0	0	1%
F5	ELALO-EpLnK1	1%	0	0	0
F6	ELALO-EpLnK5	5%	0	0	0
F7	ELALO-DBS-EpLnK1	1%	1%	0	0
F8	ELALO-Span-EpLnK1	1%	0	1%	0
F9	ELALO-HSA-EpLnK1	1%	0	0	1%
F10	ELALO-DBS-EpLnK5	5%	1%	0	0
F11	ELALO-Span-EpLnK5	5%	0	1%	0
F12	ELALO-HSA-EpLnK5	5%	0	0	1%

^a^—LnK functionalized with epoxy rings; ^b^—structure directing agents; ^c^—dibenzylidene sorbitol; ^d^—sorbitane monostearate; ^e^—12-hydroxystearic acid.

**Table 2:** Chemical shifts of C=O stretching band for ELALO-based formulations (un-cured).

Initial Systems		F1	F2	F3	F4	F6
	Absorption Band	ELALO	ELALO-DBS1	ELALO-Span1	ELALO-HSA1	ELALO-EpLnK5
ν_C=O_ (cm^−1^)		1746	1741	1744	1741	1741

**Table 3:** GF, CA, WA results.

Sample (Code and Description)		GF (%) ^a^	CA (°) ^b^	WA (%) ^c^
F1	ELALO	97.06 ± 0.49	84.24 ± 2.48	1.27 ± 0.36
F2	ELALO-DBS1	95.14 ± 0.31	81.72 ± 2.79	1.73 ± 0.20
F3	ELALO-Span1	97.32 ± 0.48	102.84 ± 1.06	0.60 ± 0.30
F4	ELALO-HSA1	96.04 ± 0.83	89.88 ± 1.14	2.11 ± 0.44
F5	ELALO-EpLnK1	95.91 ± 0.50	94.50 ± 0.96	2.03 ± 0.30
F6	ELALO-EpLnK5	79.41 ± 4,77	70.05 ± 1.58	undetermined
F7	ELALO-DBS1-EpLnK1	96.60 ± 0.56	84.02 ± 2.48	1.72 ± 0.67
F8	ELALO-Span1-EpLnK1	97.40 ± 0.46	94.28 ± 2.68	1.02 ± 0.19
F9	ELALO-HSA1-EpLnK1	97.10 ± 0.20	82.56 ± 2.62	2.30 ± 0.19
F10	ELALO-DBS1-EpLnK5	80.66 ± 0.51	88.08 ± 2.46	undetermined
F11	ELALO-Span1-EpLnK5	84.18 ± 0.66	87.18 ± 2.00	undetermined
F12	ELALO-HSA1-EpLnK5	68.05 ± 1.86	82.40 ± 3.08	undetermined

^a^—calculated gel fractions; ^b^—contact angle values; ^c^—calculated water absorption capacity.

**Table 4:** DMA results.

Sample (Code and Description)		DMA Results		
		Tg (°C)	Storage Modulus (MPa)	
			Glassy State	Rubbery State
F1	ELALO	65	8500	300
F2	ELALO-DBS1	63	4900	200
F3	ELALO-Span1	59	2900	118
F4	ELALO-HSA1	61	3100	145
F5	ELALO-EpLnK1	65	800	70
F7	ELALO-DBS1-EpLnK1	56	7300	140
F8	ELALO-Span1-EpLnK1	60	4500	160
F9	ELALO-HSA1-EpLnK1	59	5100	170

**Table 5:** TGA (N_2_ atmosphere) results.

Samples (Code and Description)		Weight Loss—Td (°C)				Tmax (°C)	Residual Mass (%, at 700 °C)
		3%	5%	10%	50%		
F1	ELALO	168	211	331	407	404	2.18
F2	ELALO-DBS1	168	211	324	407	404	2.63
F3	ELALO-Span1	164	201	313	407	404	3.98
F4	ELALO-HSA1	167	208	319	407	404	2.46
F5	ELALO-EpLnK1	178	229	333	408	407	3.84
F6	ELALO-EpLnK5	179	235	337	409	414	5.53
F7	ELALO-DBS1-EpLnK1	175	216	326	408	405	4.21
F8	ELALO-Span1-EpLnK1	172	245	335	409	408	4.50
F9	ELALO-HSA1-EpLnK1	173	232	334	410	407	4.47
F10	ELALO-DBS1-EpLnK5	176	240	332	408	406	6.25
F11	ELALO-Span1-EpLnK5	176	252	332	405	403	3.46
F12	ELALO-HSA1-EpLnK5	179	250	336	409	404	7.36

**Table 6:** XPS results.

Sample	ELALO (F1)	ELALO-Span1 (F3)	ELALO-EpLnK1 (F5)	ELALO-Span1-EpLnK1 (F8)
C/O ratio (%)	3.90	3.85	4.37	4.44

## Data Availability

Not applicable.

## Supplementary Materials

The following supporting information can be downloaded at: https://www.mdpi.com/article/10.3390/polym15020439/s1, Figure S1: General scheme showing the steps in the synthesis of the ELALO-SDA-EpLnK-type composites; Figure S2: FTIR spectra of (a) LnK, (b) EpLnK derivative; Figure S3: XPS survey spectra for (a) LnK and (b) EpLnK derivative. XPS deconvolution for high resolution O1s spectra for (c) LnK and (d) EpLnK; Tabel S1: XPS results for the LnK and its epoxy derivative; Figure S4: Idealized scheme of the chemical conversion of LnK in its epoxy derivative; Figure S5: The storage modulus vs. temperature curves for the studied ELALO-based composites.
