# Supplementary material for: Investigating the Synthesis and Characteristics of UV-Cured Bio-Based Epoxy Vegetable Oil-Lignin Composites Mediated by Structure-Directing Agents
Source: Polymers (Basel). 2023 Jan 13;15(2):439. doi: 10.3390/polym15020439 (PMC9864384; doi:10.3390/polym15020439)
Supplement: Supplementary file 1 [file polymers-15-00439-s001.zip › polymers-2072735-supplementary.pdf]

SUPPLEMENTARY FILES

Investigating the synthesis and characteristics of UV-cured bio-based epoxy vegetable oil-lignin composites mediated by structure-directing agents

Brindusa Balanuca, Raluca Sanda Komartin, Madalina Ioana Necolau, Celina Maria Damian, Raluca Stan

FIGURES

Figure S1. General scheme showing the steps in the synthesis of the ELALO-SDA-EpLnK type composites.

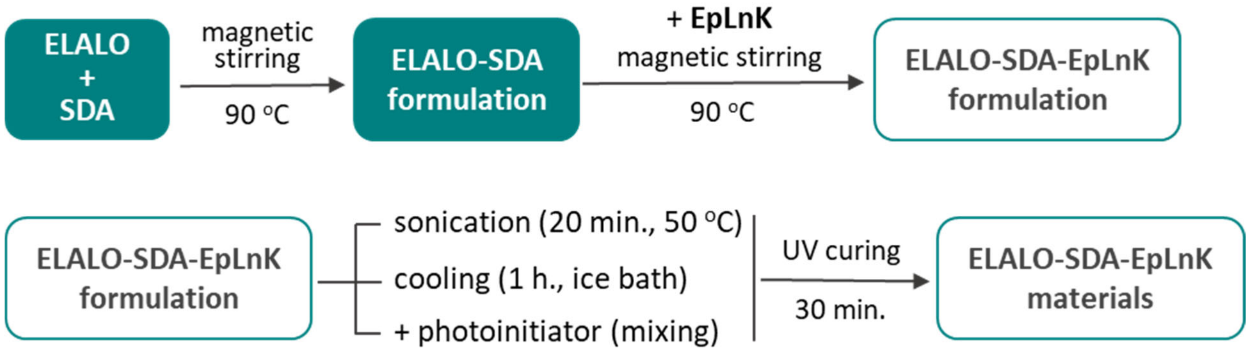

Figure S2. FTIR spectra of a) LnK, b) EpLnK derivative.

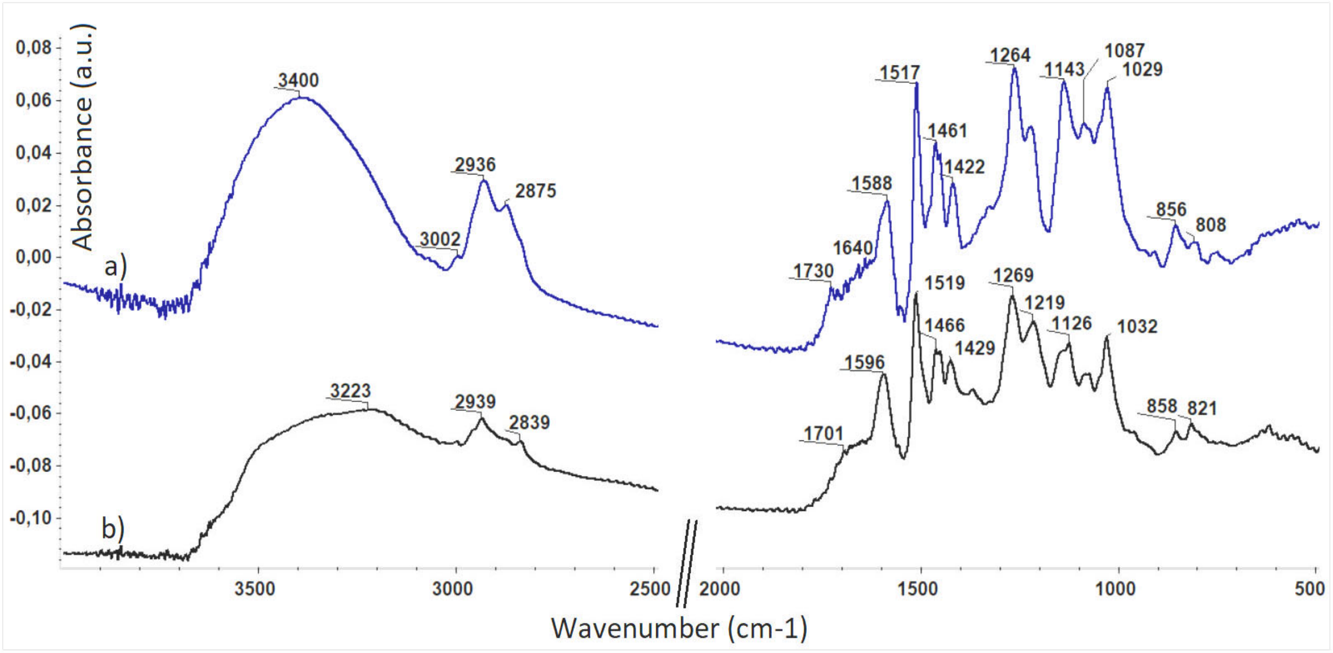

Figure S3. XPS survey spectra for a) LnK and b) EpLnK derivative. XPS deconvolution for high resolution O1s spectra for c) LnK and d) EpLnK

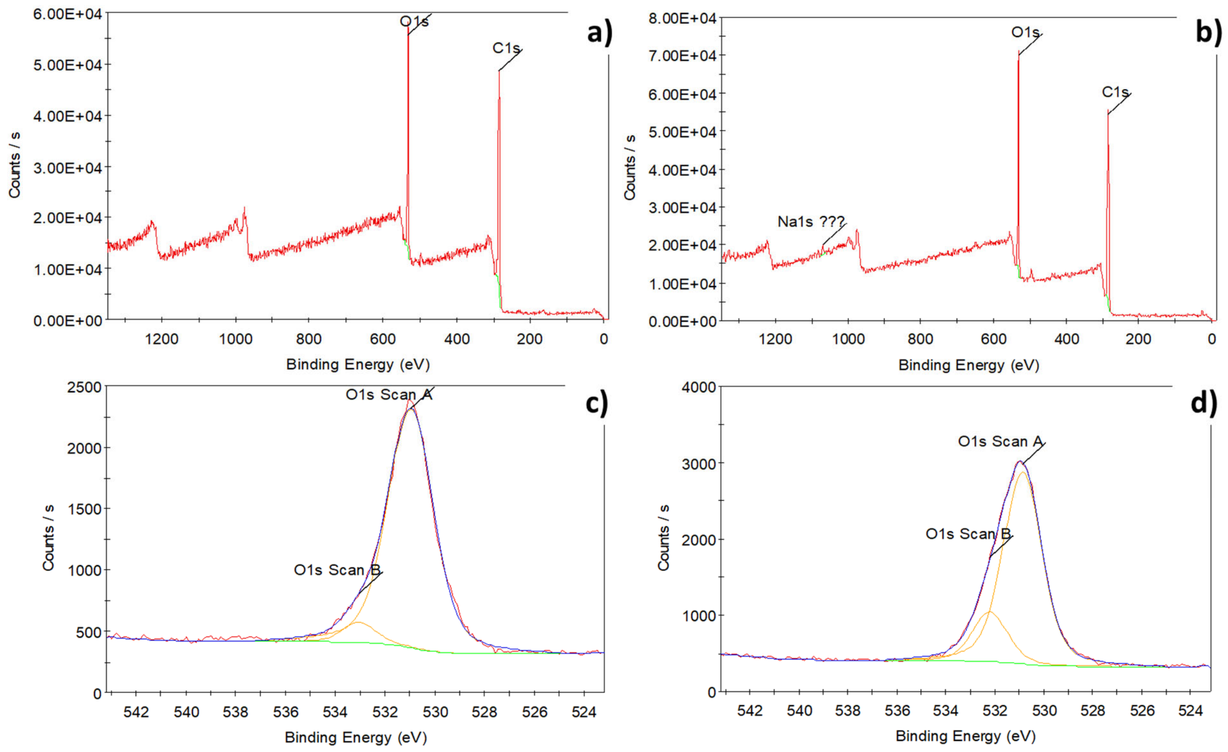

Figure S4. Idealized scheme of the chemical conversion of LnK in its epoxy derivative.

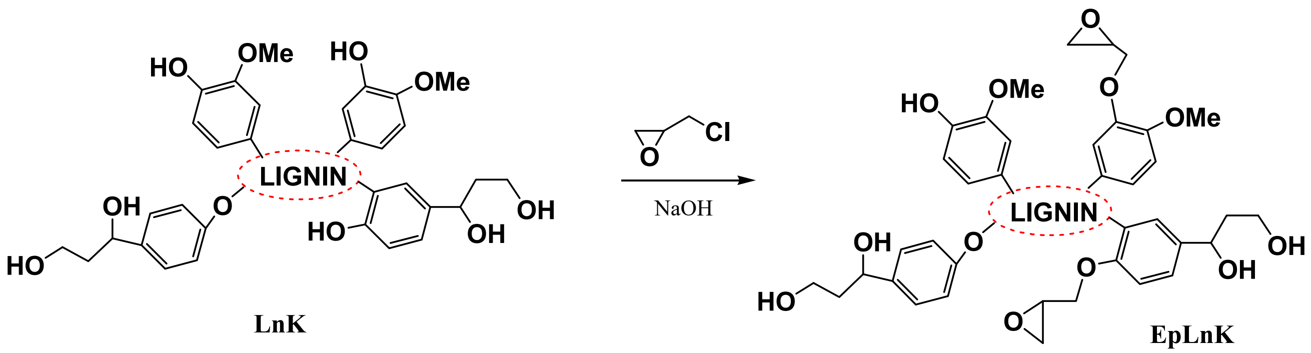

Figure S5. The storage modulus vs. temperature curves for the studied ELALO-based composites

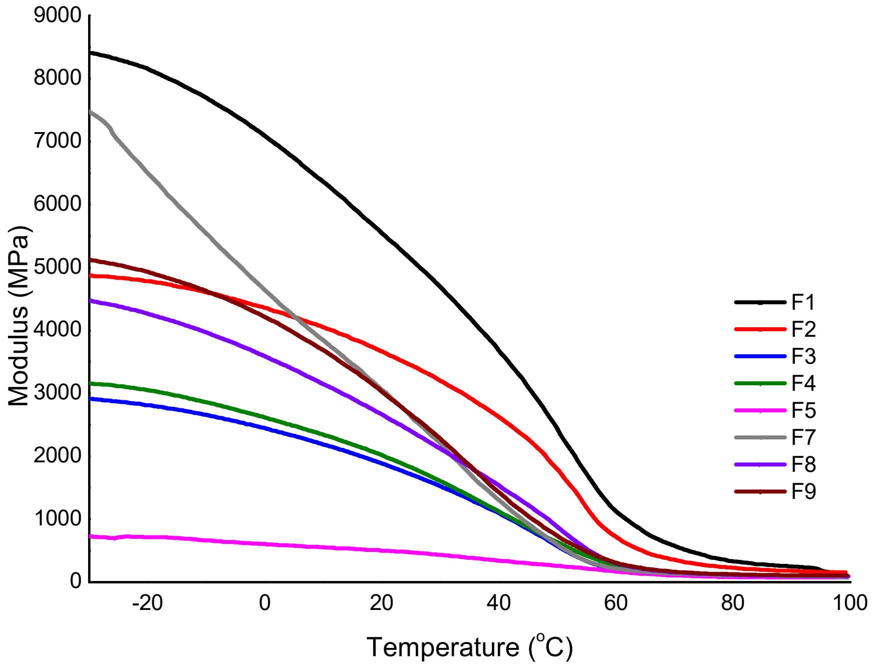

TABLES

Table S1. XPS results for the LnK and its epoxy derivative.

Sample	C ratio (%)	O ratio (%)	C/O ratio (%)
LnK	77.49	22.51	3.44
EpLnK	71.90	27.16	1.04
